# Molecular characterization and embryonic origin of the eyes in the common house spider *Parasteatoda tepidariorum*

**DOI:** 10.1186/s13227-015-0011-9

**Published:** 2015-04-28

**Authors:** Christoph Schomburg, Natascha Turetzek, Magdalena Ines Schacht, Julia Schneider, Phillipp Kirfel, Nikola-Michael Prpic, Nico Posnien

**Affiliations:** Johann-Friedrich-Blumenbach-Institut für Zoologie und Anthropologie, Abteilung für Entwicklungsbiologie, GZMB Ernst-Caspari-Haus, Georg-August-Universität Göttingen, Justus-von-Liebig-Weg 11, 37077 Göttingen, Germany; Present address: Fachbereich Medizin, Philipps-Universität Marburg, Baldingerstraße, 35032 Marburg, Germany

**Keywords:** Eye development, Spiders, Retinal determination genes, *Parasteatoda tepidariorum*

## Abstract

**Background:**

Two visual systems are present in most arthropod groups: median and lateral eyes. Most of our current knowledge about the developmental and molecular mechanisms involved in eye formation in arthropods comes from research in the model system *Drosophila melanogaster*. Here, a core set of retinal determination genes, namely, *sine-oculis* (*so*), *eyes absent* (*eya*), *dachshund* (*dac*), and the two *pax6* orthologues *eyeless* (*ey*) and *twin of eyeless* (*toy*) govern early retinal development. By contrast, not much is known about the development of the up-to-eight eyes present in spiders. Therefore, we analyzed the embryonic expression of core retinal determination genes in the common house spider *Parasteatoda tepidariorum*.

**Results:**

We show that the anlagen of the median and lateral eyes in *P. tepidariorum* originate from different regions of the non-neurogenic ectoderm in the embryonic head. The median eyes are specified as two individual anlagen in an anterior median position in the developing head and subsequently move to their final position following extensive morphogenetic movements of the non-neurogenic ectoderm. The lateral eyes develop from a more lateral position. Intriguingly, they are specified as a unique field of cells that splits into the three individual lateral eyes during late embryonic development. Using gene expression analyses, we identified a unique combination of determination gene expression in the anlagen of the lateral and median eyes, respectively.

**Conclusions:**

This study of retinal determination genes in the common house spider *P. tepidariorum* represents the first comprehensive analysis of the well-known retinal determination genes in arthropods outside insects. The development of the individual lateral eyes via the subdivision of one single eye primordium might be the vestige of a larger composite eye anlage, and thus supports the notion that the composite eye is the plesiomorphic state of the lateral eyes in arthropods. The molecular distinction of the two visual systems is similar to the one described for compound eyes and ocelli in *Drosophila*, suggesting that a unique core determination network for median and lateral eyes, respectively, might have been in place already in the last common ancestor of spiders and insects.

**Electronic supplementary material:**

The online version of this article (doi:10.1186/s13227-015-0011-9) contains supplementary material, which is available to authorized users.

## Background

**Figure 1:**
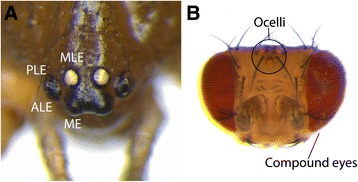
Eyes in spiders and insects. **(A)** Dorso-frontal view of the eyes of an adult specimen of *P. tepidariorum*. Adult spiders usually have eight eyes: a pair of median eyes (ME) and three pairs of lateral eyes (LE): the median (MLE), posterior (PLE) and anterior lateral eyes (ALE). Note that the PLE and ALE share a common socket in the carapace. **(B)** Dorsal view of the head of an adult specimen of *D. melanogaster*. Insects also have median and lateral eyes, but these differ in morphology from those of spiders. The median eyes are simple ocelli, whereas the lateral eyes are multi-facetted complex eyes.

Animal visual systems allow the perception of environmental information and are essential for basic behaviors like feeding, reproduction, and interaction with the environment. Bilaterians have evolved various organs to perceive visual cues ranging from simple light sensitive cells shielded by a pigment cell, for example, in the planarian *Polycelis auricularia* [1] or the trochophora larvae of the annelid *Platynereis* [2] to sophisticated eyes like compound eyes in insects and lens eyes in vertebrates [3,4]. In arthropods, two independent visual systems are present: lateral and median eyes [5] (see Figure 1). It has been proposed that these two visual systems have evolved from one primordial visual organ more than 500 million years ago [6,7].

The most comprehensive understanding of the processes involved in adult eye formation is available for insects, mainly from work performed in the fruit fly *Drosophila melanogaster.* There, the lateral compound eyes and the dorsal-median ocelli (Figure 1) originate from a few cells of the visual anlage in the dorsal head neuroectoderm in the embryo [8-10]. During the first larval instar, these cells are part of the eye-antennal imaginal disc that undergoes massive proliferation throughout larval and pupal development [11]. Within the eye-antennal imaginal disc, the two visual systems are determined in non-overlapping domains, implying that the anlagen of both visual systems develop largely independent of one another [12,13].

The retinal field (that is, the anlage of the lateral compound eyes) of the eye-antennal imaginal disc is determined on a molecular level by the action of a cascade of transcription factors that is known as the retinal determination gene network (RDGN). In summary, the *pax6* genes *eyeless* (*ey*) and *twin of eyeless* (*toy*) activate an auto-regulatory network of transcription factors involving *sine-oculis* (*so*), *eyes absent* (*eya*), and *dachshund* (*dac*) [14]. The *Drosophila* ortholog of the *six3* gene, *optix*, is involved in eye morphogenesis in an *ey*-independent manner [15] and has been linked to differentiation processes within the retinal field [16]. The core of the RDGN not only determines the retinal field that gives rise to the lateral compound eyes but also operates in the dorsal-median ocelli [17]. However, initial establishment of the ocellar primordium requires unique regulatory interactions between *engrailed* (*en*), *hedgehog* (*hh*), and *orthodenticle* (*otd*), which eventually lead to RDGN activation via *eya* in the ocelli anlagen [12,17-19]. Additionally, the RDGN genes *ey* and *dac* are only present in the determination of the lateral compound eyes [6,7,20]. These data suggest that the molecular mechanisms underlying the determination of the lateral and median eyes represent a combination of shared and unique aspects.

Intriguingly, comparative expression data accumulated over the last decades suggest that the core RDGN known from *Drosophila* might be conserved in the various different bilaterian eye types [3,21]. For instance, members of the *pax* family genes are the most widely conserved eye selector genes and appear to initiate eye development in all animals. *pax6* orthologues are expressed during eye development, for example, in Cnidarians [22-25], the lancelet *Branchiostoma floridae* [26], the polychaete *Platynereis dumerilii* [27], the ascidian *Phallusia mammillata* [28], and the onychophoran *Euperipatoides kanangrensis* [29]. However, more detailed examination of expression and/or function of RDGN genes also revealed functional differences. For instance, in the flour beetle *Tribolium* and in *Drosophila*, the *pax6* orthologues *ey* and *toy* seem to play a more dominant role during larval eye development, rather than in the adult eyes [30]. Similarly, in the American Horseshoe Crab, *Limulus polyphemus*, *pax6* does not seem to be expressed in the eye primordia during late embryogenesis, implying that it might not be involved in retinal determination [31].

**Figure 2:**
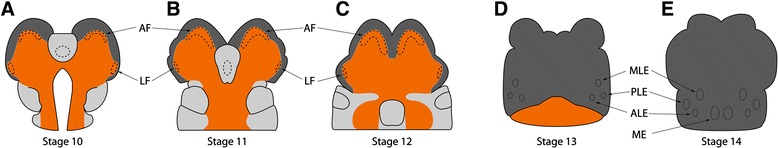
Morphogenesis of the head region of *P. tepidariorum.* Schematic drawings of embryonic heads in ventral view at stage 10 **(A)**, stage 11 **(B)**, stage 12 **(C)**, stage 13 **(D)**, and stage 14 **(E)**. Stages were defined after [57]. The non-neurogenic ectoderm is shown in gray; the neurogenic ectoderm is shown in orange. The non-neurogenic ectoderm at the anterior rim of the head lobes (dark gray) gradually overgrows the neurogenic ectoderm. The anterior (AF) and lateral furrow (LF) in the brain primordium are indicated. In the non-neurogenic head ectoderm, the primordia of the eyes are also indicated: ALE, anterior lateral eyes; PLE, posterior lateral eyes; MLE, median lateral eyes; ME, median eyes.

In terms of visual system evolution, chelicerates represent an interesting arthropod group because various different eye types have evolved in this class. Horseshoe crabs (Xiphosura) possess large compound lateral eyes, but their median eyes are highly reduced [32,33]. Other chelicerate groups, for example, scorpions [34] and spiders [35] have a varying number of simple lateral eyes and one pair of simple median eyes (that may be reduced). In, for example, harvestmen (Opiliones), only a pair of simple median eyes are present, but lateral eyes are entirely missing [36]. Mites (Acari) may have a pair of median eyes and one to three pairs of lateral eyes, but most Acari species are lacking eyes altogether [37]. Spiders usually have four pairs of eyes: (1) one pair of median eyes (ME), which lack a light-reflecting tapetum and usually are the largest eyes and thus the main optical system [35], and (2) three pairs of lateral eyes, which usually have a light-reflecting tapetum. In adult spiders, the innermost pair of lateral eyes is often situated directly behind the median eyes and they are therefore sometimes called posterior median eyes [35]. However, we prefer the term median lateral eyes (MLE) to clearly denote them as lateral eyes. Depending on their location, the other two lateral eyes are called anterior lateral eyes (ALE) and posterior lateral eyes (PLE) (Figures 1A and 2D,E).

The arthropod ground plan includes both median and lateral eyes as two separate visual systems. However, in extant arthropod groups, one of the two systems is usually the dominant visual system, strongly suggesting that a single visual system is largely sufficient. For instance, in insects, the main visual organs are the lateral eyes (compound eyes), whereas the median eyes are simple lens eyes (ocelli) with only poor visual capacities. The ocelli have instead acquired novel functions, for example, in flight stability control [38], or have been entirely reduced in many insect groups [39]. In contrast, in most spiders, the median eyes represent the main visual system, while the lateral eyes mostly visualize movement [35]. Insects and spiders have thus used different evolutionary strategies for their visual systems.

However, so far only little is known about eye development in spiders and the molecular genetic mechanisms that govern their formation. Therefore, we have isolated homologues of several retinal determination genes known from *Drosophila* in the spider *Parasteatoda tepidariorum* and present here an analysis of their expression patterns throughout head development. *P. tepidariorum* represents an excellent model system for comparative studies due to its short life cycle, access to offspring throughout the year, and various established methods like *in situ* hybridization and RNA interference [40]. We show that the anlagen of the median eyes originate from an anterior median position in the developing head, while the lateral eyes develop from a more lateral position. Intriguingly, the lateral eyes develop from a single field of cells that splits into the three individual lateral eyes during late embryonic development. Besides differences in the embryonic origin, we identified a unique combination of determination gene expression in the anlagen of the lateral and median eyes, respectively. This molecular distinction of the two visual systems is similar to the one described for compound eyes and ocelli in *Drosophila*, suggesting that a unique core determination network for median and lateral eyes, respectively, might have been in place already in the last common ancestor of spiders and insects.

## Methods

### Animal culture and gene cloning

Embryos were obtained from our laboratory stock in Göttingen. The embryos were staged as previously published [41]. The following already published eye determination genes were used in this study: *pax6.1* (FM945394.1), *dac1* (FM945397.1), *six3.1* (AB605265.1), *six3.2* (AB605266.1), and *otd1* (AB096074.1). The sequences of additional genes were identified by BLAST search of the sequences from *D. melanogaster* against the *P. tepidariorum* transcriptome [41]: *pax6.2* (KP725068), *so1* (KP725069), *so2* (KP725070), *eya* (KP725071), *dac2* (KP725072), *otd2* (KP725073), and peropsin (KP725074). Paralogs were named “1” and “2,” respectively, according to their sequence similarity to the *D. melanogaster* sequence. Total RNA was extracted from a mix of all embryonic stages using TRIzol® (Life Technologies, Carlsbad, CA, USA). cDNA was synthesized from total RNA with the SMARTer™ PCR cDNA Synthesis Kit (Clontech, Mountain View, CA, USA). Gene-specific cDNA fragments were amplified with primers designed with Primer3 [42] (Additional file 1: Table S1) and cloned into the pCR®II vector using the TA Cloning® Kit Dual Promoter (Invitrogen, Life Technologies, Carlsbad, CA, USA).

### Pylogenetic analysis of spider Opsin homologues

For phylogenetic analysis of Opsin homologues, candidate sequences were identified by BLAST search of the *D. melanogaster* Rhodopsin protein sequences against the transcriptome of *P. tepidariorum* [41]. In addition to *P. tepidariorum* sequences, Opsin protein sequences for a multiple sequence alignment were obtained from published sources [43-46] (see also Additional file 2: Table S2). Multiple sequence alignment was performed with MUSCLE version 3.8.31 [47,48]. The pylogenetic tree was inferred using the parallel version of MrBayes 3.2.4 [49-52] with default settings for the likelihood model and the priors for the phylogenetic model. The amino-acid substitution model was set to “Blosum” that was determined as the model best suited for the input data by the MrBayes program.

### *In situ* staining and imaging

*In situ* hybridization and nuclear staining with SYTOX® Green were performed as described before [53,54] with minor modifications. Instead of PBST, we used MABT for rehydration and washing steps after the hybridization, as well as commercially available blocking agent from Roche (Basel, Switzerland) (2% in MABT). Images were taken with Leica M205 FA binocular (Leica Microsystems, Wetzlar, Germany) equipped with a QImaging MicroPublisher 5.0 RTV camera (QImaging, Surrey, Canada) and UV light. Stacks of nuclear SYTOX® stainings were taken with a Zeiss LSM 510 microscope (Zeiss, Oberkochen, Germany). Images were corrected for color values and brightness with Adobe Photoshop image processing software (version 12.0).

## Results

### Morphogenesis of the non-neurogenic ectoderm in the embryonic head

*P. tepidariorum* has one pair of median eyes and three pairs of lateral eyes (Figure 1A). Similar to several other members of the spider family Theridiidae, the anterior (ALE) and posterior lateral eyes (PLE) are located very close to each other [55], and in *P. tepidariorum* adults, they even share a common socket in the carapace (Figure 1A).

**Figure 3:**
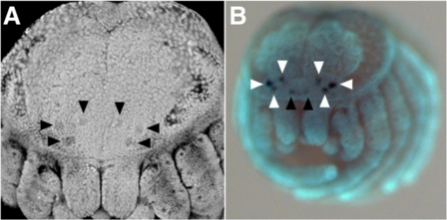
Markers for eye primordia at the final embryonic stages. **(A)** Confocal microscopy image of nuclear SYTOX® staining of a stage 14 embryo in frontal view. The primordia of the lateral eyes are visible as shallow pits in the head region (arrowheads). **(B)** Expression of *Pt-peropsin* at stage 14 marks not only the lateral eye primordia (white arrowheads) but also the median eye primordia (black arrowheads).

In *P. tepidariorum*, the formation of the brain begins at stage 9 with the invagination of clusters of neuroblast precursors from the anterior neurogenic ectoderm [56,57] (orange tissue in Figure 2A to D). Shortly after the onset of neuroblast invagination, the first signs of brain differentiation are visible as deep grooves in the head neurogenic ectoderm near the anterior rim of the germ band (anterior furrow, AF in Figure 2A to C) and oval pits near the lateral edge of the head lobes (lateral furrow, LF in Figure 2A to C). The rim of the head lobes itself (shown in dark gray in Figure 2) comprises only non-neurogenic ectoderm and starts to overgrow the neurogenic ectoderm of the head at stage 11/12 (Figure 2B,C). Until stage 13, the non-neurogenic ectoderm originating from the anterior and lateral rim of the head lobes has almost fully overgrown the neurogenic ectoderm and thus covers the developing brain almost completely (Figure 2D). At stage 14, the brain primordium is fully covered by the non-neurogenic ectoderm (Figure 2E). The first morphological signs of the lateral eye primordia can be detected as shallow grooves in the non-neurogenic ectoderm from stage 13 onwards (Figures 2D,E and 3A). The median eyes are morphologically only visible in postembryonic stages [57].

### *Pt-peropsin* is expressed in all eye primordia at late embryonic stages

Since the eye primordia in *P. tepidariorum* are morphologically visible only late during embryonic and postembryonic development, we sought to identify a molecular marker that prefigures the eye primordia. We identified one homologue of the peropsin group and four homologues from the group of r-Opsins (Additional file 3: Figure S1). We cloned these five opsins and analyzed their expression throughout late embryonic development (Figure 3B and not shown). *Pt-peropsin* is the only *opsin* homologue that is detectable at embryonic stages. It is expressed at stage 14 in three spots on either side of the prosomal shield (Figure 3B, white arrowheads). These expression domains coincide morphologically with the indentations of the lateral eyes in the epidermal tissue (Figure 3A, black arrowheads). Additionally, *Pt-peropsin* is expressed in two spots in the anterior median region of the prosomal shield (Figure 3B, black arrowheads). In this median position, the two median eyes will develop during first instar stages [57]. In summary, we identify *Pt-peropsin* as embryonic molecular marker for developing eyes in the spider *P. tepidariorum.*

### Spider homologues of *Drosophila* retinal determination genes

We have isolated homologues of key components of the retinal determination gene network known from *Drosophila*, and have studied their expression in all eye primordia of *P. tepidariorum*. In the following, we give a detailed account of the expression of all studied genes during the developmental stages 10 to 14, which comprise the time span between the formation of the non-neurogenic ectoderm at the anterior and lateral rim of the head lobes, the overgrowth of the brain anlage, and the development of the primordia of the lateral and median eyes within the non-neurogenic ectoderm.

### Homologues of *pax6*

**Figure 4:**
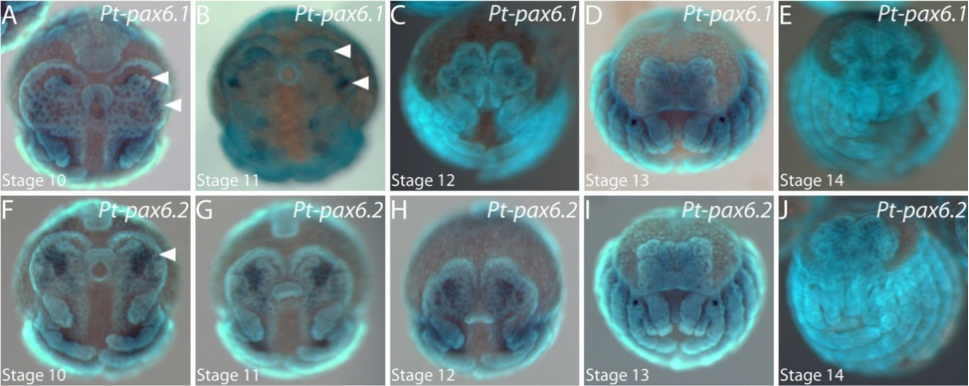
Embryonic expression of *pax6* homologues in the head of *P. tepidariorum.* **(A** to **E)** Expression of *Pt-pax6.1* during head development. A large connected domain in the brain anlage is denoted by arrowheads in A and B. **(F** to **J)** Expression of *Pt-pax6.2* during head development. A transversal stripe of expression is denoted by the arrowhead in F. All embryos are shown in frontal aspect. The developmental stage is indicated in each panel in the lower left corner.

We identified two *pax6* orthologues in the transcriptome of *P. tepidariorum* (Samadi et al., submitted) [41]. At stages 10 and 11, *Pt-pax6.1* is expressed in a narrow domain in the neurogenic ectoderm directly adjacent to the anterior and lateral furrows (Figure 4A,B, arrowheads). Throughout stages 12 to 14 while the non-neurogenic ectoderm overgrows the brain primordium, the *Pt-pax6.1* expression domains remain in the neurogenic ectoderm (Figure 4C to E). From stage 13 onwards, *Pt-pax6.1* expression can only be detected as diffuse signal in the brain anlage that is already fully covered by the non-neurogenic ectoderm (Figure 4D,E).

*Pt-pax6.2* is expressed in a transversal stripe across the neurogenic ectoderm in the head lobes and a smaller domain, which surrounds the lateral furrow (Figure 4F, arrowhead). At stages 11 and 12, the transversal domain condenses, while the expression surrounding the lateral furrow ceases (Figure 4G,H). During non-neurogenic ectoderm overgrowth at stages 13 and 14, *Pt-pax6.2* shows a diffuse expression in the underlying brain anlagen (Figure 4I,J). Note that the non-neurogenic ectoderm outside of the anterior and lateral furrows is free of *Pt-pax6.1* and *Pt-pax6.2* expression throughout the embryonic stages analyzed.

### Homologues of *six1/sine-oculis*

**Figure 5:**
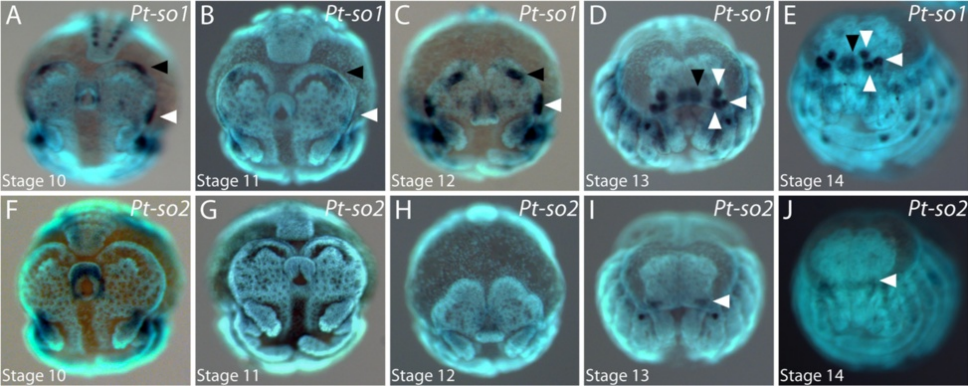
Embryonic expression of *sine-oculis* homologues in the head of *P. tepidariorum.* **(A** to **E)** Expression of *Pt-so1* during head development. Two separate domains in the non-neurogenic ectoderm are denoted by arrowheads in A to C. Expression in the lateral eye primordia is indicated by arrowheads in D. **(F** to **J)** Expression of *Pt-so2* during head development. Late expression in the median eye primordia is denoted by an arrowhead in I and J. All embryos are shown in frontal aspect. The developmental stage is indicated in each panel in the lower left corner.

**Figure 6:**
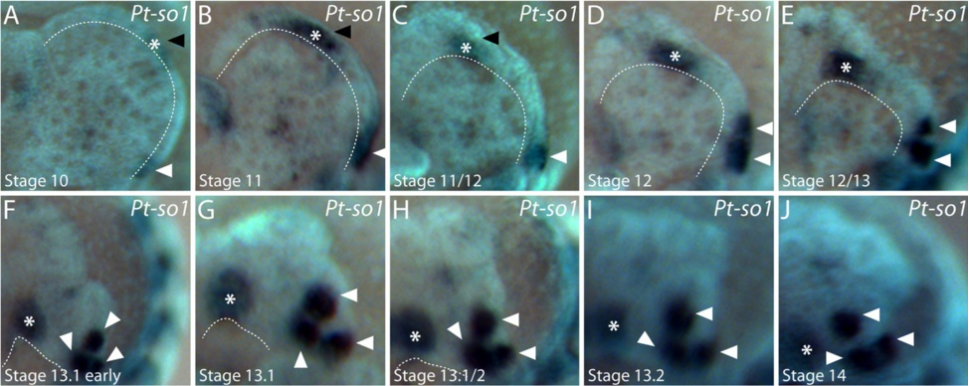
Dynamics of *sine-oculis1* expression during brain overgrowth. All panels show the left half of the head lobes in frontal view **(A** to **J)**. Expression in the primordia of the median eyes is denoted by the asterisk, expression in the lateral eye primordia is indicated by white arrowheads in all panels. The dotted line shows the leading edge of the non-neurogenic head ectoderm that overgrows the brain anlage. The developmental stage is indicated in each panel in the lower left corner.

We identified two orthologues of the *six1/so* gene in the transcriptome of *P. tepidariorum* (Samadi et al., submitted) [41]. In the head anlagen at stages 10 and 11, *Pt-so1* expression is observed in a region around the stomodeum and in two domains at the rim of the head lobes next to the anterior and lateral furrows, respectively (arrowheads in Figure 5A,B; Figure 6A to C). While the non-neurogenic ectoderm overgrows the neurogenic ectoderm of the head from stage 12 onwards, the anterior *Pt-so1* expression domain always remains at the leading edge of the non-neurogenic ectoderm throughout development (black arrowhead in Figure 5C; asterisk in Figure 6A to H; the leading edge is indicated by the dotted line). Thus, the domain is relocated from the anterior rim of the head lobe to the anterior rim of the prospective carapace (black arrowhead in Figure 5D; asterisk in Figure 6H), which is also the location where *Pt-peropsin* expression is observed in the primordia of the median eyes (compare Figure 5D,E with Figure 3B). When the non-neurogenic ectoderm has fully overgrown the brain anlage, *Pt-so1* is diffusely expressed in the primordia of the median eyes (black arrowhead in Figure 5E; asterisk in Figure 6I,J). By contrast, the lateral expression domain does not change its relative location and does not follow the leading edge of the overgrowing non-neural ectoderm (Figure 5C to E; white arrowheads in Figure 6). In stage 12, this domain becomes slightly bipartite (Figure 6D) and then splits into two separate domains at the transition between stage 12 and stage 13 (Figure 6E). At early stage 13, the more posterior domain splits once more (Figure 6F) and buds off another domain (Figure 6G), which however stays in close vicinity to its parental domain (Figure 6H to J).

*Pt-so2* is expressed in the labrum at stage 10 (Figure 5F), but is not expressed in the brain anlage or the non-neurogenic ectoderm during stages 10 to 12 (Figure 5F to H). However, once the brain anlage is fully overgrown by the non-neurogenic ectoderm, a new expression domain of *Pt-so2* arises at stage 13 in the non-neurogenic ectoderm close to the posterior-lateral edge of the head lobes (Figure 5I, arrowhead) and persists throughout stage 14 in the primordia of the anterior lateral eyes (Figure 5J, arrowhead).

### Homologue of *eyes-absent*

**Figure 7:**
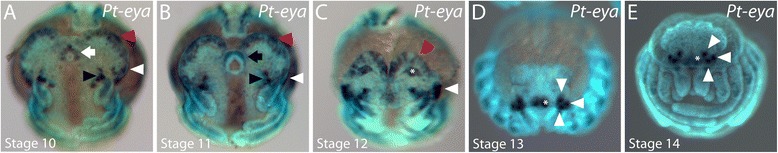
Embryonic expression of the *eyes-absent* homologue in the head of *P. tepidariorum.* **(A** to **E)** Expression of *Pt-eya* during head development. A dynamic expression domain along the rim of the head lobes is denoted by arrowheads in A to C. Expression in the lateral eye primordia is indicated by white arrowheads in D and E. The black arrowheads in A and B point to expression at the base of the chelicerae. The arrows denote expression in the stomodeum **(A)** and in the labrum **(B)**. All embryos are shown in frontal aspect. The developmental stage is indicated in each panel in the lower left corner.

For *eya*, we only found a single orthologue in the transcriptome (Samadi et al., submitted) [41]. At stages 10 and 11, *Pt-eya* is expressed along the edge of the head lobes (red and white arrowheads in Figure 7A,B). No expression is detected in the anterior and lateral furrows. Additional expression domains are present around the stomodeum (white arrow in Figure 7A), in the developing labrum (black arrow in Figure 7B) and in spots at the base of the chelicerae (black arrowhead in Figure 7A, B). At stage 11, *Pt-eya* expression ceases at a lateral position thus dividing the expression along the rim into two separate domains (red and white arrowheads in Figure 7B). When the non-neurogenic ectoderm starts to grow over the brain anlagen, *Pt-eya* expression at the rim of the leading edge becomes stronger (asterisk in Figure 7C) and ends up in the primordia of the medial eyes (asterisk in Figure 7D,E). The remaining anterior expression becomes patchy (red arrowhead in Figure 7C) and then vanishes entirely in early stage 13 (Figure 7D and not shown). The lateral portion of the rim domain increases in expression level (white arrowhead in Figure 7C) and then separates first into two and finally into three pairs of dots in the primordia of the lateral eyes (white arrowheads in Figure 7D,E and not shown).

### Homologues of *dachshund*

**Figure 8:**
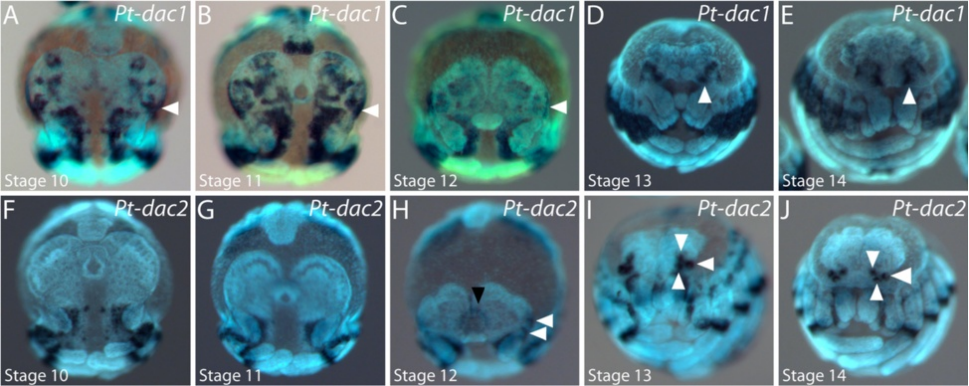
Embryonic expression of *dachshund* homologues in the head of *P. tepidariorum.* **(A** to **E)** Expression of *Pt-dac1* during head development. The arrowhead in A to C points to expression at the lateral edge of the head lobes. The arrowhead in D and E points to expression in the primordia of the anterior lateral eyes. **(F** to **J)** Expression of *Pt-dac2* during head development. Expression in the lateral eye primordia is denoted by white arrowheads in H to J. The black arrowhead in H denotes expression anterior to the labrum. All embryos are shown in frontal aspect. The developmental stage is indicated in each panel in the lower left corner.

We found two orthologues of the *dac* gene in the transcriptome of *P. tepidariorum* (Samadi et al., submitted) [41,58]. *Pt-dac1* shows a complex pattern in the neurogenic ectoderm of the head and all body segments that increases in complexity with the advancement of neural maturation. At stage 10, there are up to three distinguishable domains of *Pt-dac1* expression in the brain anlage (Figure 8A). In addition, *Pt-dac1* is expressed at the posterior lateral edge of the head lobes (arrowhead in Figure 8A to C). The expression pattern in the brain anlage becomes more complex in stage 11 (Figure 8B) but then almost completely ceases at stage 12 (Figure 8C). The lateral expression, however, remains strong and ends up in the primordia of the anterior lateral eyes and in cells at a lateral position on the developing carapace (arrowhead in Figure 8D,E).

*Pt-dac2* is not expressed in the head lobes before stage 12 (Figure 8F,G). At stage 12, expression of *Pt-dac2* appears anterior to the labrum and in two distinct domains in the lateral head (Figure 8H). During stages 13 and 14, all primordia of the lateral eyes express *Pt-dac2* very strongly (white arrowheads in Figure 8I,J).

### Homologues of *six3/optix*

**Figure 9:**
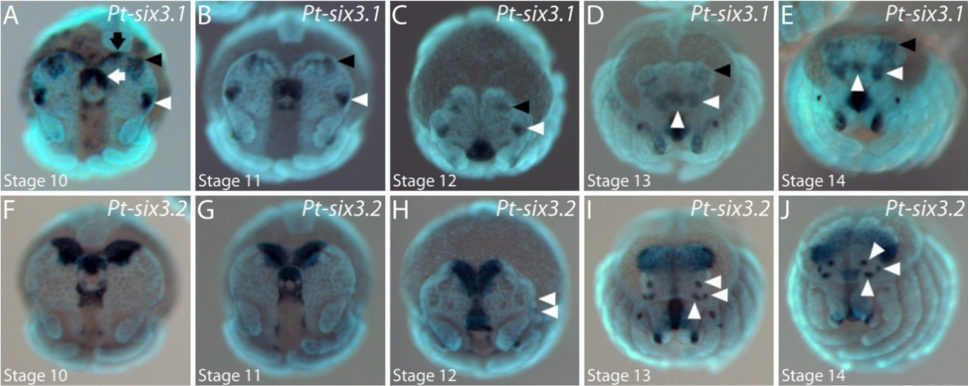
Embryonic expression of *six3* homologues in the head of *P. tepidariorum.* **(A** to **E)** Expression of *Pt-six3.1* during head development. Two separate domains in the brain anlage are denoted by arrowheads in A to C. The black arrow in A denotes expression along the anterior end of the head lobes. Expression in the labrum is denoted by a white arrow in A. The white arrowheads in D and E point to the tripartite expression in the brain primordium that develops from the earlier lateral expression domains (white arrowheads in A to C). **(F** to **J)** Expression of *Pt-six3.2* during head development. Expression in the lateral eye primordia is denoted by arrowheads in H to J. All embryos are shown in frontal aspect. The developmental stage is indicated in each panel in the lower left corner.

We identified two *optix/six3* orthologues in *P. tepidariorum* (Samadi et al., submitted) [41]. At stage 10, *Pt-six3.1* is expressed in the labrum (white arrow in Figure 9A), at the anterior rim of the head lobes (black arrow in Figure 9A) and in an anterior (black arrowhead in Figure 9A) and a lateral spot (white arrowhead in Figure 9A) in the neurogenic ectoderm. The expression at the anterior rim of the head lobes vanishes during stage 11 (Figure 9B). The other domains remain throughout stages 11 and 12 (arrowheads in Figure 9B,C) and become covered by the non-neurogenic ectoderm. At stage 13, the *Pt-six3.1* expression in the neurogenic ectoderm is completely overgrown by the non-neurogenic ectoderm (Figure 9D). The formerly lateral expression of *Pt-six3.1* becomes more complex during the stages 13 and 14 (white arrowheads in Figure 9D, E). Thus, although *Pt-six3.1* shows a dynamic expression pattern in the neurogenic ectoderm, this gene is not expressed in the overlaying non-neurogenic ectoderm and not in the primordia of the eyes.

**Figure 10:**
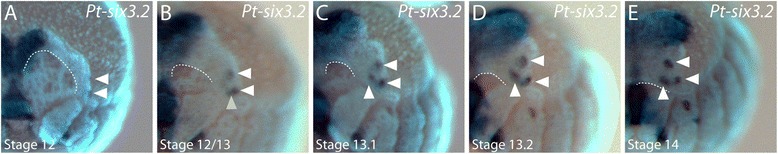
Dynamics of *six3.2* expression during brain overgrowth **(A to E)**. All panels show the left half of the head lobes in frontal view. Expression in the lateral eye primordia is indicated by arrowheads in all panels. The dotted line shows the leading edge of the non-neurogenic head ectoderm that overgrows the brain anlage. The developmental stage is indicated in each panel in the lower left corner.

At stages 10 and 11, *Pt-six3.2* is expressed in a wedge-shaped domain in the anterior median region of the head, in the labrum, and in the posterior stomodeum (Figure 9F,G). The anterior median domain is fully overgrown by the non-neurogenic ectoderm during further development (Figure 9H to J). Two expression domains appear *de novo* at the lateral edge of the head lobes (Figures 9H and 10A), of which the posterior one splits into two domains (Figure 10B) resulting in three distinct domains of *Pt-six3.2* expression (arrowheads in Figure 9I,J; Figure 10C to E). Thus, all primordia of the lateral eyes express *Pt-six3.2*. Note that the primordia of the median eyes do not express *Pt-six3.2*; the blurred staining in Figure 9J at the position where the median eyes develop is located in the foregut primordium beneath the head tissue.

### Homologues of *orthodenticle*

**Figure 11:**
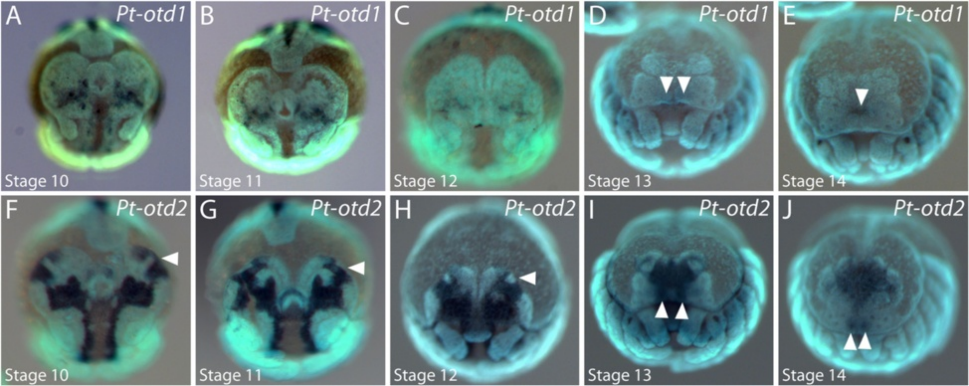
Embryonic expression of *orthodenticle* homologues in the head of *P. tepidariorum.* **(A** to **E)** Expression of *Pt-otd1* during head development. The arrowheads in D and E point to expression in the brain anlage underneath the non-neurogenic ectoderm. **(F** to **J)** Expression of *Pt-otd2* during head development. Arrowheads in F to J denote *Pt-otd2* expression in the non-neurogenic ectoderm, which ends up in the median eye anlagen. All embryos are shown in frontal aspect. The developmental stage is indicated in each panel in the lower left corner.

Two orthologues of the *otd* gene have been identified in *P. tepidariorum* (Samadi et al., submitted) [41,58,59]. *Pt-otd1* is expressed in two large, patchy domains in the developing brain on each side of the posterior head lobes throughout stages 10 to 12 (Figure 11A to C). During stages 13 and 14, the non-neurogenic ectoderm grows over the *Pt-otd1-*positive cells, which end up in the center of the developing brain posterior to the median eye anlagen (arrowheads in Figure 11D,E).

At stages 10 and 11, *Pt-otd2* is expressed in a broad domain in the center of the developing brain and in two connected domains at the rim of the head lobes (white arrowheads in Figure 11F,G). This entire expression is very dynamic during further development and finally covers most of the forming brain (Figure 11H to J). Parts of the anterior rim domain move posteriorly with the overgrowing non-neurogenic ectoderm during stages 12 to 14 (white arrowheads in Figure 11G to J), resulting in *Pt-otd2* expression in two small clusters of cells in the anterior-median non-neurogenic ectoderm that correspond to the median eye primordia (white arrowheads in Figure 11J).

## Discussion

### Embryonic origin of median and lateral eyes

Although the eyes become morphologically visible only in late embryonic stages (in the case of the lateral eyes) or even in postembryonic stages (in the case of the median eyes) [57], the primordia of all eyes can be identified much earlier based on their expression of conserved retinal determination genes.

The primordia of the median eyes are defined in non-neurogenic tissue that is at first located at the anterior rim of the head lobes, but then moves with the leading edge of the overgrowing tissue and finally the median eyes are situated at the anterior rim of the developing carapace. This movement of the median eye primordia can be followed best using the expression of *Pt-so1* because this gene is expressed in the median eye primordia from their initial allocation at the anterior rim of the head lobe until their final location at the anterior rim of the carapace (Figure 6).

The lateral eye primordia are also formed in non-neurogenic ectoderm, but at the lateral rim of the head lobes and they do not move with the leading edge of the overgrowing non-neural ectoderm. Intriguingly, our data suggest that at a molecular level all lateral eyes are initially specified as a single field of cells on each side of the head lobes. Differentiation into three separate lateral eyes then occurs later by splitting of the original uniform eye field. The first splitting gives rise to the median lateral eyes and the primordium of the posterior and anterior lateral eyes. In a second splitting event, the latter primordium then separates into the posterior and the anterior lateral eyes. It is tempting to speculate whether this composite lateral eye primordium is a remnant of a phylogenetically older complex lateral eye primordium [5,60]. Indeed, basal chelicerates (for example, *L. polyphemus*) have complex lateral eyes [32], several fossil arthropod groups have complex lateral eyes (for example, Trilobites) [61], and multi-facetted complex eyes are widespread in the crustaceans and insects [62]. This suggests that the formation of the lateral eyes via a common eye field that is subsequently subdivided into smaller eye units represents the plesiomorphic condition of the lateral visual system in the arthropods.

### Every eye type expresses a unique combination of transcription factors

**Figure 12:**
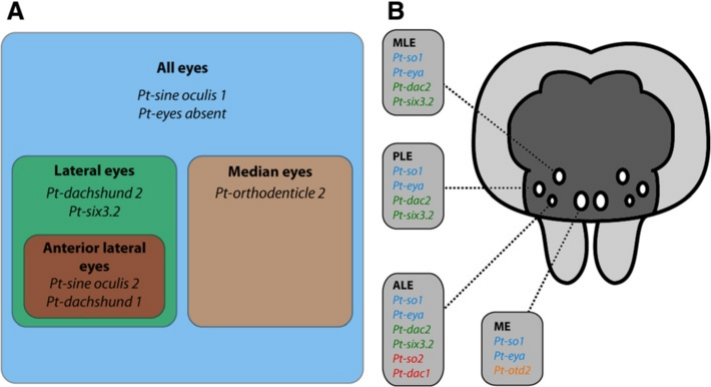
Summary of the expression of eye patterning genes in eye primordia of *P. tepidariorum.* **(A)** The studied genes distinguish the different eyes. Apart from general eye genes (blue), other genes mark the median eyes (orange) and the lateral eyes (green) and even distinguish the anterior lateral eyes (red). **(B)** The four eye pairs of spiders express their specific combinations of eye developmental genes. ALE, anterior lateral eyes; PLE, posterior lateral eyes; MLE, median lateral eyes; ME, median eyes.

Our data show that most of the key genes of the retinal determination gene network known from *Drosophila* have a conserved expression in the eyes of *P. tepidariorum*. All eye primordia express *Pt-so1* and *Pt-eya*, and thus, these genes might be involved in specifying eye identity in general (Figure 12A,B). This is in accordance with eye development in *Drosophila* where the transcription factor So and the co-activator Eya form a protein complex to activate downstream targets within the retinal determination network in the developing compound eyes [63] and in the ocelli [19,64,65]. In the lateral compound eyes, the So/Eya complex activates the transcription factor *dac* that mediates eye specification [20,63]. However, ocelli development is not affected by loss of function mutants of *dac* [20]. Intriguingly, we find expression of both *dac* orthologues in *P. tepidariorum* exclusively in the lateral eyes (Figure 12A,B), suggesting that the involvement of *dac* in eye determination might be specific to lateral eyes not only in *Drosophila* but also in arthropods in general. Similarly, *optix/six3* is involved in compound eye formation in *Drosophila* [15], while a potential function during ocelli formation remains to be elucidated. Of the two *six3* genes in *P. tepidariorum*, only *Pt-six3.2* is expressed in the anlagen of the lateral eyes (Figure 12A,B). Therefore, expression of *Pt-dac2* and *Pt-six3.2* is specific to the lateral eyes and might specify lateral eye identity in general. The median eyes do not express these lateral eye factors, but express *Pt-otd2* as a unique marker for median eyes. Also in *Drosophila*, it has been shown that *otd* is essential to define the ocelli primordia [12,18,19], while a function in the compound eyes is only evident during late differentiation stages [66,67]. Not only the median and lateral eyes seem to be specified by different genetic mechanisms in the spider, but also among the lateral eyes developmental genetic differences exist. The anterior lateral eyes are specifically characterized by expression of *Pt-so2* and *Pt-dac1* (Figure 12A,B), while we do not find gene expression that is specific to either the median or posterior lateral eyes. Of course, the latter two eyes could be further distinguished by the expression of genes that were not included in our present study.

In summary, we find hints that a separation of the core eye determination network into two separate functional units governing median and lateral eye formation, respectively, might have been present already in the ancestral arthropod and thus may predate the split between chelicerates and insects. While in insects and spiders, the anlagen of median eyes are defined by the presence *otd* and a lack of So/Eya-mediated *dac* activation, lateral eye development relies on the action of Dac and Six3 in addition to the So/Eya complex that is necessary for the formation of all eyes (Figure 12A,B). Additional molecular diversification of lateral eyes in spiders might be facilitated by the presence of paralogous eye determination genes.

### The role of Pax6 during spider eye development

Intriguingly, we do not detect late expression of the two *pax6* orthologues in any of the eye primordia of *P. tepidariorum*. A similar observation has been made in the American Horseshoe Crab *L. polyphemus* where late embryonic eye anlagen are free of *pax6* expression [31]. This result is unexpected since Pax6 is required for eye development not only in insects but also, for example, in mammals [68-70]. In *Limulus*, early *pax6* expression was not studied but qPCR results imply an activity prior to the analyzed late stages [31]. Hence, it is likely that Pax6 is required early during anterior development to specify the future eye anlagen. This hypothesis is supported by *Pt-pax6.1* expression at the anterior rim of stage 6/7 germ disc embryos of *P. tepidariorum* (not shown), suggesting that this gene might be essential for the early specification of the non-neurogenic ectoderm at the anterior and lateral rim of the developing head lobes. Whether this early anterior expression of *Pt-pax6.1* is involved in the activation of further eye determination genes remains to be elucidated.

### A potential role of peropsins during eye development

We identified a peropsin orthologue in *P. tepidariorum* that clusters with other chelicerate and vertebrate sequences (Additional file 3: Figure S1). Peropsins are expressed in the retina of several vertebrates [71,72] and amphioxus [73] where it is proposed to function as a retinal photoisomerase. Clear protostome peropsin orthologues have only been identified in spiders [74,75] and in the American horseshoe crab *L. polyphemus* [76]. We detect *Pt-peropsin* expression in the embryonic anlagen of lateral and median eyes, suggesting an early involvement in visual system development or function. Intriguingly, an early function during ocular development has been proposed for peropsins in mice and humans where expression can be detected already during early embryonic stages [72]. Hence, the accessibility of *P. tepidariorum* development and the availability of functional tools represents an excellent model to further investigate a potential role of peropsins in eye development.

## Conclusions

The two eye types in spiders, lateral and median eyes, develop from two different areas in the non-neurogenic ectoderm. Although morphological signs of the eye primordia appear only in the final stages of embryogenesis or later, on the molecular level, the eyes are specified much earlier. Apart from factors that are expressed in all eyes, there are also factors that distinguish the lateral eyes and the median eyes. In addition, among the lateral eyes, the anterior ones are patterned differently from the remaining lateral eyes. The primordia of the median eyes are initially allocated at the anterior rim of the head lobes from where they move to their final location at the anterior rim of the carapace with the non-neural ectoderm that overgrows the underlying brain anlage. All lateral eyes on each side of the head develop from a common eye field, thus providing evidence for the notion that the individual lateral eyes in adult spiders are actually evolutionary remnants of a composite lateral eye. It will be interesting to study the formation and specification of the lateral eyes in spiders in more depth to compare it to the development of the facetted eyes in insects.

## Additional files

Additional file 1: Table S1. Table with the primer sequences used to amplify the genes of interest from cDNA. Additional file 2: Table S2. Names and accession numbers of sequences used in the phylogenetic analysis of the *P. tepidariorum* Peropsin sequence. Additional file 3: Figure S1. Phylogenetic tree of opsin sequences with the human Melatonin Receptor 1A as outgroup. Sequences were obtained from published literature (Additional file 2: Table S2). The tree was built using amino-acid sequences. Branch values are the posterior probabilities of Bayesian likelihood. The *P. tepidariorum* Peropsin sequence clusters to the Peropsin group with vertebrates and *C. salei*, while the other *P. tepidariorum* sequences analyzed cluster with the r-Opsin group.

## Abbreviations

ALE: anterior lateral eyes
AF: anterior furrow
BLAST: Basic Local Alignment Search Tool
bp: base pairs
cDNA: complementary DNA
LF: lateral furrow
ME: median eyes
MLE: median lateral eyes
PLE: posterior lateral eyes
qPCR: quantitative polymerase chain reaction
RDGN: retinal determination gene network
