# Supplementary figures and images for: Molecular characterization and embryonic origin of the eyes in the common house spider Parasteatoda tepidariorum
Source: EvoDevo. 2015 Apr 28;6:15. doi: 10.1186/s13227-015-0011-9 (PMC4450840; doi:10.1186/s13227-015-0011-9)

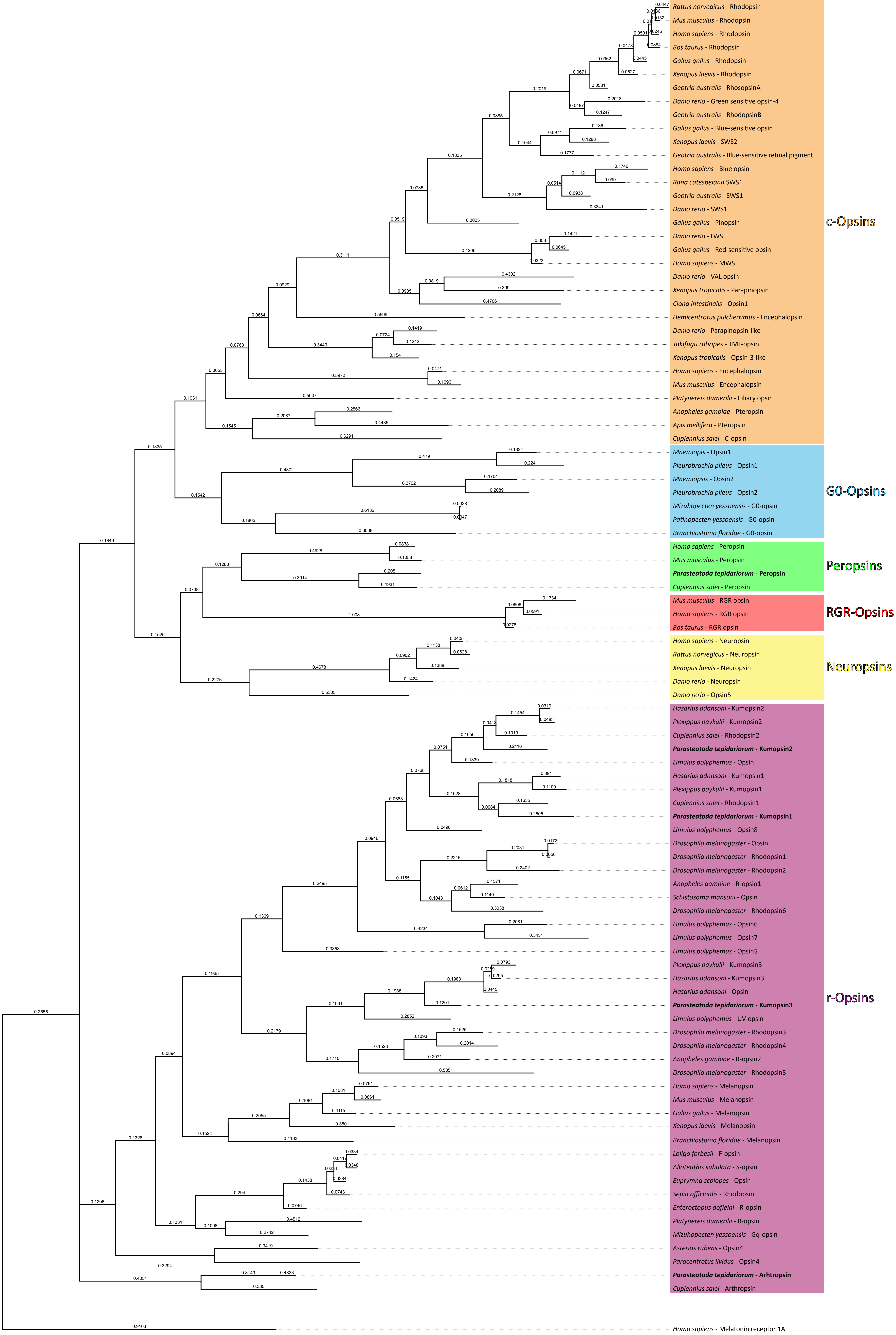

Supplement: Additional file 3: Figure S1. — Phylogenetic tree of opsin sequences with the human Melatonin Receptor 1A as outgroup. Sequences were obtained from published literature (Additional file 2: Table S2). The tree was built using amino-acid sequences. Branch values are the posterior probabilities of Bayesian likelihood. The P. tepidariorum Peropsin sequence clusters to the Peropsin group with vertebrates and C. salei, while the other P. tepidariorum sequences analyzed cluster with the r-Opsin group. [file 13227_2015_11_MOESM3_ESM.pdf]
